# Copy number variants underlie major selective sweeps in insecticide resistance genes in *Anopheles arabiensis*

**DOI:** 10.1371/journal.pbio.3002898

**Published:** 2024-12-05

**Authors:** Eric R. Lucas, Sanjay C. Nagi, Bilali Kabula, Bernard Batengana, William Kisinza, Alexander Egyir-Yawson, John Essandoh, Sam Dadzie, Joseph Chabi, Arjen E. Van’t Hof, Emily J. Rippon, Dimitra Pipini, Nicholas J. Harding, Naomi A. Dyer, Chris S. Clarkson, Alistair Miles, David Weetman, Martin J. Donnelly

**Affiliations:** 1 Department of Vector Biology, Liverpool School of Tropical Medicine, Liverpool, United Kingdom; 2 National Institute for Medical Research, Amani Research Centre, Muheza, Tanzania; 3 Department of Biomedical Sciences, University of Cape Coast, Cape Coast, Ghana; 4 Department of Parasitology, Noguchi Memorial Institute for Medical Research, University of Ghana, Accra, Ghana; 5 Biology Centre of the Czech Academy of Sciences, Institute of Entomology, České Budějovice, Czech Republic; 6 Big Data Institute, Li Ka Shing Centre for Health Information and Discovery, University of Oxford, Oxford, United Kingdom; 7 Wellcome Sanger Institute, Hinxton, Cambridge, United Kingdom; University of Exeter, UNITED KINGDOM OF GREAT BRITAIN AND NORTHERN IRELAND

## Abstract

To keep ahead of the evolution of resistance to insecticides in mosquitoes, national malaria control programmes must make use of a range of insecticides, both old and new, while monitoring resistance mechanisms. The outdoor-biting malaria vector *Anopheles arabiensis* is of increasing concern for malaria transmission because it is apparently less susceptible to many indoor control interventions, yet knowledge of its mechanisms of resistance remains limited. Furthermore, comparatively little is known in general about resistance to non-pyrethroid insecticides such as pirimiphos-methyl (PM), which are crucial for effective control in the context of globally high resistance to pyrethroids. We performed a genome-wide association study to determine the molecular mechanisms of resistance to the pyrethroid deltamethrin (commonly used in bednets) and PM (widespread use for indoor spraying), in *An*. *arabiensis* from 2 regions in Tanzania. Genomic regions of positive selection in these populations were largely driven by copy number variants (CNVs) in gene families involved in metabolic resistance. We found evidence of a new gene cluster involved in resistance to PM, identifying a strong selective sweep tied to a CNV in the carboxylesterase genes *Coeae2g - Coeae6g*. Using complementary data from another malaria vector, *An*. *coluzzii*, in Ghana, we show that copy number at this locus is significantly associated with PM resistance. Similarly, for deltamethrin, resistance was strongly associated with a novel CNV allele in the *Cyp6aa* / *Cyp6p* cluster (*Cyp6aap*_Dup33). Against this background of metabolic resistance, resistance caused by mutations in the insecticide target sites was very rare or absent. Mutations in the pyrethroid target site *Vgsc* were at very low frequency in Tanzania, yet combining these samples with 3 *An*. *arabiensis* individuals from West Africa revealed a startling evolutionary diversity, with up to 5 independent origins of *Vgsc*-995 mutations found within just 8 haplotypes. Thus, despite having been first recorded over 10 years ago, *Vgsc* resistance mutations in Tanzanian *An*. *arabiensis* have remained at stable low frequencies. Overall, our results provide a new copy number marker for monitoring resistance to PM in malaria mosquitoes, and reveal the complex picture of resistance patterns in *An*. *arabiensis*.

## Abstract 2

Understanding the evolution of insecticide resistance in malaria mosquitoes is crucial for effective malaria control. A genome-wide association study of resistance in Anopheles arabiensis to two key insecticides reveals high copy number at the carboxylesterase gene cluster Coeae2g - Coeae6g as a novel genetic cause of resistance to pirimiphos-methyl.

## 1. Introduction

The evolution of insecticide resistance in disease vectors threatens effective control of vector-borne diseases such as malaria [1–4], in the same way as antibiotic resistance is jeopardising the effective treatment of bacterial infections. In large parts of Africa, malaria-transmitting mosquitoes have already developed resistance to the most widely used class of public health insecticides, pyrethroids [5,6]. In response to this, other insecticides have been deployed, such as indoor residual spraying (IRS) with the organophosphate pirimiphos-methyl (PM) [7]. For the effectiveness of these interventions to be sustained, resistance to the new compounds needs to be anticipated and monitored.

The 2 main mechanisms of insecticide resistance are target site resistance, where the protein targeted by the insecticide is mutated to reduce insecticide binding, and metabolic resistance, where increased levels, or affinity, of metabolic enzymes accelerates the breakdown or sequestration of insecticides and their by-products [8]. For pyrethroids, the target site is the voltage-gated sodium channel (*Vgsc*), with mutations in codon 1014 (numbering from *Musca domestica*; codon 995 in *Anopheles gambiae*) providing resistance in a wide range of species [9–13], while metabolic resistance is often provided by elevated activity of cytochrome P450s, particularly from the *Cyp6* and *Cyp9* families [12,14–17]. The target site of organophosphates such as PM is acetylcholinesterase (*Ace1)*, with resistance typically being provided by a mutation in codon 119 (numbering from *Torpedo californica*; codon 280 in *An*. *gambiae*) in combination with gene duplication [18–21], while metabolic resistance often results from elevated expression of carboxylesterases [12,22,23]. Most pyrethroids can also be degraded or sequestered by esterases, although the extent of this differs by insecticide and by species [24]. In mosquitoes, esterases are not typically associated with resistance to pyrethroids [25], and any evidence of association so far has been correlative [26,27].

Metabolic resistance can be achieved through a much broader range of mutations than target site resistance, making it harder to identify causative alleles. However, increases in the number of genomic copies of a gene, known as copy number variants (CNVs), are a tractable form of mutation that have repeatedly been implicated in metabolic resistance to both pyrethroids [28,29] and organophosphates [23,30–32].

In the *Anopheles gambiae* species complex (which includes the major malaria vectors *An*. *gambiae s*.*s*., *An*. *coluzzii*, and *An*. *arabiensis*), high levels of PM resistance have already been detected in parts of West Africa, where it is primarily driven by the *Ace1*-280S single-nucleotide polymorphism (SNP) and a CNV in *Ace1* [21], as well as esterase CNVs [33]. In contrast, East Africa has fewer reported cases of PM resistance (Fig A in S1 Text) and an absence of *Ace-1* resistance mutations in any malaria vector species. It is therefore crucial to investigate populations showing early evidence of PM resistance to understand the nature of this resistance and unravel the genetic mechanisms that underlie it, to better monitor incipient resistance across the region.

While indoor-based interventions such as IRS and insecticide-treated nets (ITNs) have successfully reduced numbers of *An*. *gambiae s*.*s*. in East Africa, outdoor-biting species such as *An*. *arabiensis* have been less affected [34]. Resistance levels in *An*. *arabiensis* are typically lower than in indoor biting species, probably due to their reduced exposure to insecticides, but are nonetheless appreciable to some active ingredients [35–38]. This is a cause for concern since *An*. *arabiensis* is a significant vector of malaria [34], and in some areas the primary vector [39–41]. In East Africa, resistance in *An*. *arabiensis* has been reported to deltamethrin [35,37], a pyrethroid widely used in bednets, and PM [42]. This provides an ideal opportunity to study the genomics of resistance in this species, both for established (deltamethrin) and recently introduced (PM) insecticides.

**Fig 1:**
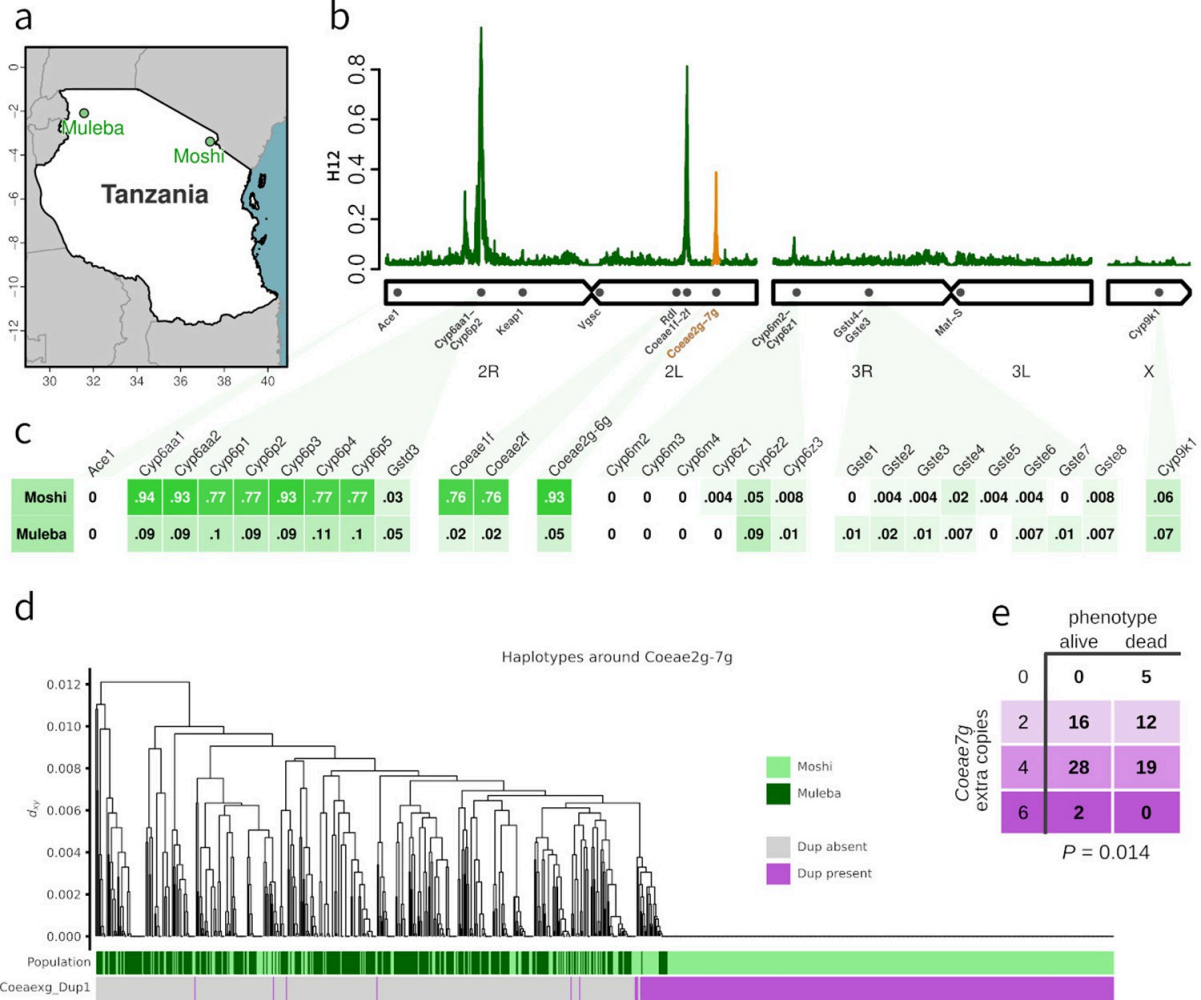
(**a**) Map of sampling locations. GPS coordinates are given in Data S1. (**b**) Genome-wide H_12_ calculated in 2,000 SNP windows in samples from Moshi, showing peaks in selection signal around *Cyp6aa/Cyp6p*, *Coeae1f-2f*, and *Coeae2g-7g* (highlighted in orange). (**c**) Proportion of samples carrying a CNV in key metabolic genes (columns) in each of our 2 sample sites (rows). Mosquitoes in Moshi had >70% frequency of CNVs in the *Coeae1f-2f* and *Coeae2g-6g* genes (this CNV allele does not include *Coeae7g*), as well as *Cyp6aa/Cyp6p*. (**d**) Haplotype clustering of the genomic region around *Coeae2g-7g* in Moshi and Muleba. Haplotypes bearing the CNV allele *Coeaexg*_Dup1 were almost perfectly associated with the large swept cluster seen on the right, indicating that the CNV is likely to be driving the selective sweep. (**e**) In *An*. *coluzzii* from Korle-Bu, Ghana, copy number of *Coeae7g* was significantly associated with resistance to PM (*P* = 0.014 after controlling for CNV in *Ace1*). Panels a, b, c, and d were produced using scripts found at https://github.com/vigg-lstm/GAARD_east (https://zenodo.org/records/13898157. Panel a: manuscript_figures/Fig1a.r using shape file downloaded from https://spatial.faoswalim.org/layers/geonode:Africa_Adm0_Country, panel b: manuscript_figures/Fig1b.r using underlying data at selection_analysis/H12_Moshi*.csv, panel c: CNV_analysis/CNV_analysis_tanzania.r using underlying data at CNV_analysis/Ag1000G_CNV_data/v3.7_1246-VO-TZ-KABULA-VMF00185/modal_CNVs/modal_copy_number_arabiensis.csv, panel d: CNV_analysis/sweeps/Coeaexg_CNV_haplotypes.ipynb using data at CNV_analysis/Ag1000G_CNV_data/v3.7_1246-VO-TZ-KABULA-VMF00185/target_regions_analysis/focal_region_CNV_table.csv and accessing Ag1000G data directly from the cloud using the malariagen_data Python package (malariagen.github.io/malariagen-data-python/latest/Ag3.html)). Panel arrangements and highlighting were performed in Inkscape. CNV, copy number variant; PM, pirimiphos-methyl; SNP, single-nucleotide polymorphism.

As part of the Genomics for African *Anopheles* Resistance Diagnostics (GAARD) project, we are using large-scale whole genome sequencing to investigate the genomics of insecticide resistance in key regions of Africa. Here, we investigated resistance in *An*. *arabiensis* from 2 contrasting regions of Tanzania (Fig 1A). Moshi is an elevated area with extensive rice and sugarcane plantation and associated irrigation [43], with the possibility of resistance developing due to exposure to insecticides used on crops. Muleba is an area that has been the site of vector control trials, and where mosquitoes have thus been exposed to a range of public health insecticides, including PM [3,44,45]. We conducted a genome-wide association study (GWAS) of resistance to deltamethrin and PM in these 2 populations.

## 2. Results

### 2.1. Bioassays

Preliminary bioassays conducted in Moshi and Muleba indicated the presence of deltamethrin resistance in both locations, but PM resistance only in Moshi (S1 Data). In Moshi, 24-h mortality to deltamethrin ranged from 53% at 0.5× the WHO diagnostic concentration, to 73% at 2.5×, then 99% to 100% at each of 5×, 7.5×, and 10×. Mortality was slightly higher in Muleba, with 64% mortality at 0.5×, 71% at 1×, 97% at 2.5×, then 100% at 5× and above. For PM, in Moshi mortality ranged from 58% at 0.5× to 86% at 1×, then 100% at 2×. In contrast, there was no evidence of any resistance to PM in Muleba, with 100% mortality even at 0.5× concentration. These bioassays were conducted on mosquitoes identified morphologically as members of the *An*. *gambiae* species complex (*An*. *gambiae s*.*l*.). Molecular species identification performed on a subset of these confirmed that all samples in Muleba (196 out of 196) and nearly all in Moshi (382 out of 384) were *An*. *arabiensis*, with the final 2 in Moshi being *An*. *gambiae s*.*s*. All further analyses were performed on *An*. *arabiensis* only.

### 2.2. Overview of genomic data

Samples for this study were sequenced as part of the MalariaGEN Vector Observatory release Ag3.7 (https://www.malariagen.net/data), which produces SNP and CNV calls, as well as phased haplotype calls. Data were obtained from 467 individual female mosquitoes across 3 sample sets (Table 1). The phenotype of each individual was defined by whether they were alive after exposure to a high dose of insecticide (“resistant”) or dead after exposure to a lower dose (“susceptible”), thus providing strong phenotypic separation between phenotypes. Exposure conditions to generate the distinct phenotype classes were calculated separately for each location and are reported in S1 Data.

**Table 1:** Number of samples sequenced in each of the 3 sample sets (rows), after removal of siblings and contaminated samples.

Location	Insecticide	Final *N* Dead/alive
Moshi	Delta	51/80
Moshi	PM	69/82
Muleba	Delta	81/81

Delta, deltamethrin; PM, pirimiphos-methyl.

We calculated kinship using the KING statistic [46] pairwise across all 467 samples to identify close kin pairs (full sibs), which would be nonindependent data points in an association study. This resulted in the identification of 18 sib groups containing a total of 38 individuals (16 groups of 2 siblings, 2 groups of 3 siblings). All sib groups contained only samples from a single location (2 groups from Moshi, 16 groups from Muleba). Depending on the analysis (see Methods), we either discarded all but one randomly chosen individual per sib group per sample set (thus removing 19 samples) or performed permutations in which we varied which individuals were discarded in each sib group. We found that 4 of the samples had universally high relatedness values to all other samples in the data set. Closer inspection revealed that these samples had elevated heterozygosity due to cross-sample contamination (Fig B in S1 Text), which causes artefactual inflation of KING values. We therefore removed these 4 samples from all analyses.

A principal component analysis (PCA) of the samples based on SNP genotypes indicated genetic differentiation between our 2 sampling sites, but no other evidence of defined clusters in the first 4 principal coordinates (Fig C in S1 Text).

### 2.3. Signals of selection point to a new carboxylesterase gene cluster

We first identified regions of the genome undergoing recent positive selection by performing genome-wide H_12_ scans (Figs 1B and Fig D in S1 Text), combining the data from the deltamethrin and PM experiments. Signals of selection in *Anopheles gambiae* are often the result of insecticidal pressures [47], but do not indicate which insecticides are responsible for a given signal, and thus constitute a preliminary analysis of the data to identify regions of potential interest.

In both Moshi and Muleba, the strongest signal of selection across the genome is centred on the cluster of *Cyp6aa*/*Cyp6p* genes on chromosome 2R, a region repeatedly associated with resistance to deltamethrin. H_1x_ analysis [48] indicated that the signals in Moshi and Muleba in this genomic region are shared, with the same mutations underlying the selection signal in both regions (Fig D in S1 Text).

In Moshi, 2 peaks in H_12_ were also found on chromosome 2L (Fig 1B). The first was centred on the carboxylesterases *Coeae1f* (AGAP006227) and *Coeae2f* (AGAP006228), which have been implicated in resistance to PM in West Africa [33]. The second peak was centred on another carboxylesterase cluster, *Coeae2g* (AGAP006723)—*Coeae7g* (AGAP006728), which has not previously been associated with resistance.

### 2.4. Copy number variants are associated with resistance to PM and deltamethrin

CNVs in the carboxylesterase genes *Coeae1f* and *Coeae2f* have recently been associated with resistance to PM in *An*. *gambiae s*.*s*. from Ghana, and have been found in *An*. *arabiensis* from Tanzania [33]. In our samples, we found the previously identified *Coeaexf*_Dup2 CNV allele in 74% of samples from Moshi, and 1% of samples from Muleba (Figs 1B and Fig E in S1 Text). We also found 5 other CNV alleles in this cluster, all at low frequencies ranging from 0.4% to 4% of samples in either Moshi or Muleba (Fig E in S1 Text). We identified selective sweeps (large groups of highly similar haplotypes, indicative of rapid spread through positive selection) by constructing hierarchical clustering trees of the phased haplotype data in each population. Haplotype clustering of the *Coeae1f-2f* region indicated the presence of 2 selective sweeps, with the more common of the 2 swept clusters being associated with *Coeaexf*_Dup2 (Fig F in S1 Text). However, *Coeaexf*_Dup2 was present in only a subset of the haplotypes in the sweep, indicating that this CNV likely appeared on this haplotype after it began sweeping. There was no association of *Coeae1f-2f* copy number in Moshi with resistance to either deltamethrin (*P* = 0.96 and *P* = 0.83 for *Coeae1f* and *Coeae2f*, respectively) or PM (*P* = 0.28 and *P =* 0.29). Because the lack of association with PM resistance was unexpected, given the role that *Coeae1f-2f* CNVs play in PM resistance in Ghana, we investigated whether this could be due to lack of statistical power in our data, but this was not the case. We ran simulations assuming that presence/absence of the CNV provided a similar effect size of resistance as was previously found in *An*. *gambiae* from Ghana [33] and found that we had 88% power to detect the effect in our data.

To explore the selection signal which we identified in the carboxylesterase genes *Coeae2g*-*Coeae7g*, we also investigated CNVs in this genetic region (Figs 1C and Fig E in S1 Text). We found a CNV covering the genes *Coeae2g* - *Coeae6g*, which we call *Coeaexg*_Dup1 (Fig G in S1 Text), at very high frequency (94% of samples) in Moshi (where resistance to PM is prevalent) and lower frequency (6%) in Muleba (where mosquitoes are completely susceptible to PM). Haplotype clustering indicated the presence of 1 major swept haplotype cluster in this genomic region, which corresponded almost exactly to the presence of the CNV (Fig 1D), implying that the CNV is driving the selective sweep. Copy number of this CNV was highly variable and could reach very high values (the median copy number among samples carrying the CNV was 8, with a maximum of 28 extra copies). However, we found no significant association of copy number in Moshi with resistance to either deltamethrin (*P* = 0.94) or PM (*P* = 0.38).

In a previous GAARD study in West Africa, we had identified a signal of association with PM resistance in *An*. *coluzzii* on chromosome 2L in the regions of 36898300–37190282 and 37558030–37585789 [49]. These regions did not include the *Coeae2g-7g* gene cluster itself (2L,37282290–37295276) and the signals had not been prioritised for further investigation. In light of the current observation, we revisited the West African GAARD data and searched for CNVs in *Coeae2g-7g*. We found CNVs at low frequency in *An*. *gambiae* populations from Madina and Obuasi (Ghana) and Baguida (Togo), as well as in *An*. *coluzzii* from Avrankou (Benin). In contrast, in *An*. *coluzzii* from Ghana (Korle-Bu), we found high CNV frequencies comparable to those in Moshi (94% of samples), although the copy number of these CNVs was lower than in Moshi (median: 4, max: 6 extra copies). The CNVs in West Africa were less clearly defined, in terms of discordant reads that could precisely distinguish CNV alleles and identify start and end points, but they encompassed a larger genomic region than the *An*. *arabiensis* allele, including *Coeae7g* (Fig G in S1 Text). There was a significant association of *Coeae2g-7g* CNVs with resistance to PM in Korle-Bu. Copy number of all carboxylesterases within the CNV (*Coeae2g - Coeae7g*) were highly cross-correlated, and thus when the copy number of one gene was included, the addition of other genes did not further improve the model. The gene with the strongest association was *Coeae7g*, both with the marker alone in the model (*P* = 0.031) and after inclusion of copy number in *Ace1* (*Ace1 P* = 3 × 10^−10^; *Coaea7g P* = 0.014, Fig 1E). However, we note that all the genes in the cluster were significantly associated with PM resistance in a model containing only that gene and *Ace1* (e.g., *Coeae6g*, *P* = 0.02). From these data alone, it is therefore uncertain which proteins in this cluster are most important for conferring resistance.

CNVs in the *Cyp6aa/Cyp6p* region were at much higher frequency in Moshi (77% to 94% depending on the gene) compared to Muleba (9% to 11%, Fig 1B). Only 4 samples, all from Moshi, carried one of the 30 CNV alleles previously identified in phase 3 of the Ag1000G project (Fig E in S1 Text). The remaining CNVs comprised 7 new alleles which we named *Cyp6aap*_Dup31—*Cyp6aap*_Dup37. The most common alleles were *Cyp6aap*_Dup33 (found in 77% of Moshi samples and 7% of Muleba samples) and a pair of CNVs in complete linkage with each other, *Cyp6aap*_Dup31 and *Cyp6aap*_Dup32 (33% of Moshi samples). We investigated whether the haplotype undergoing a selective sweep in this genomic region was associated with these CNVs. A haplotype clustering tree of the region showed a large selective sweep, shared between Moshi and Muleba (cluster 1 in Fig F in S1 Text). Both the *Cyp6aap*_Dup31/32 and *Cyp6aap*_Dup33 CNVs formed separate subgroups within this haplotype cluster, indicating that they likely appeared on this haplotype after it began sweeping. A second, smaller, selective sweep was also seen (cluster 2 in Fig F in S1 Text). Few haplotypes belonged to neither sweep, indicating that mutations in *Cyp6aa*/*Cyp6p* that have been under positive selection are now almost fixed in the population.

Copy number of genes in the *Cy6paa/Cyp6p* region was significantly associated with resistance to deltamethrin in Muleba, but not in Moshi. As with *Coeae2g-7g*, all *Cyp6aa/Cyp6p* genes were highly correlated with each other in terms of copy number, and thus it is impossible to confidently determine from these data which gene was of primary importance. Generalised linear models of phenotype association found that *Cyp6p2* and *Cyp6p3* showed the strongest association of copy number with resistance (*P* = 8 × 10^−6^, compared to *P* = 2 × 10^−4^ for *Cyp6aa1*), and after inclusion of one of these genes in the model, no other genes provided further significant improvement. We then investigated the 2 CNV alleles (*Cyp6aap*_Dup31/32 and *Cyp6aap*_Dup33) separately, as well as the swept haplotype. As with overall copy number, *Cyp6aap*_Dup33 was strongly associated with resistance in Muleba (*P* = 3 × 10^−5^, *Cyp6aap*_Dup31/32 was absent in Muleba), but not Moshi (*P* = 0.1 for *Cyp6aap_Dup33*, *P* = 0.48 for *Cyp6aap*_Dup31/32). In both locations, the large swept haplotype itself was nearly, but not quite, significantly associated with resistance (*P* = 0.084 and *P* = 0.08 in Moshi and Muleba, respectively), suggesting that in Muleba, *Cyp6aap*_Dup33 provides resistance over and above that which might be conferred by other mutations on its haplotype background.

### 2.5. Known resistance SNPs

Target site resistance mutations were very rare in our samples. In *Ace1* (the target site of PM), the resistance SNP *Ace1*-280S was completely absent. We found 5 other non-synonymous SNPs in *Ace1* with a minor allele count of at least 5 in our PM sample set from Moshi, but all were low frequency and none were significantly associated with resistance (Table A in S1 Text).

The only recognised resistance SNP present in *Vgsc* (target site of deltamethrin) was *Vgsc*-995F (2 out of 564 haplotypes in Moshi, 0 out of 324 haplotypes in Muleba). We also found the SNP *Vgsc*-1507I, which has previously been found on the haplotype background of *Vgsc*-995F in *An*. *coluzzii* from Guinea [50], in a single sample from Moshi, which did not carry *Vgsc*-995F. The most common target site resistance mutation was in *Rdl*, the target site for the organochlorine dieldrin, with *Rdl*-296S found in 2% of haplotypes from Moshi. In contrast to these target site mutations, the metabolic resistance SNP *Cyp4j5*-43F was fixed for the mutant allele in all our samples.

We further investigated the 2 *Vgsc*-995F mutants to determine whether they were introgressed from, or of similar evolutionary origin to, the same mutation found in other populations of *An*. *gambiae* and *An*. *arabiensis*. We performed haplotype clustering in the *Vgsc* gene as in previous work [47,50], combining our samples with all 2,784 samples from phase 3 of the Ag1000G. This includes 368 *An*. *arabiensis* individuals, but only 3 of which (i.e., 6 haplotypes) are from West Africa. All 3 are from Burkina Faso and have resistance mutations in *Vgsc* (2 cases of the leucine to phenylalanine mutation *Vgsc*-995F and 4 cases of the leucine to serine mutation *Vgsc*-995S). We found little geographical structure among *Vgsc* haplotypes, with the 6 haplotypes from Burkina Faso being interspersed among East African samples (Fig H in S1 Text). Despite there being only 4 *Vgsc*-995F haplotypes in the entire sample set, these were found in 3 different parts of the haplotype tree, indicating that between them they represent 3 different evolutionary origins of the mutation, with the 2 mutant haplotypes in our Tanzanian samples being of different origins, and the 2 Burkinabè mutants together forming a third origin. The 4 *Vgsc*-995S haplotypes from Burkina Faso formed 2 clusters which, while being close together in the tree, were separated by wild-type haplotypes. None of the *Vgsc*-995 mutations in *An*. *arabiensis* clustered with *An*. *gambiae s*.*s*. or *An*. *coluzzii* haplotypes, indicating that these are likely to have originated within *An*. *arabiensis* rather than having introgressed from these other species.

### 2.6. Windowed measures of differentiation/selection to identify genomic regions associated with resistance

**Fig 2:**
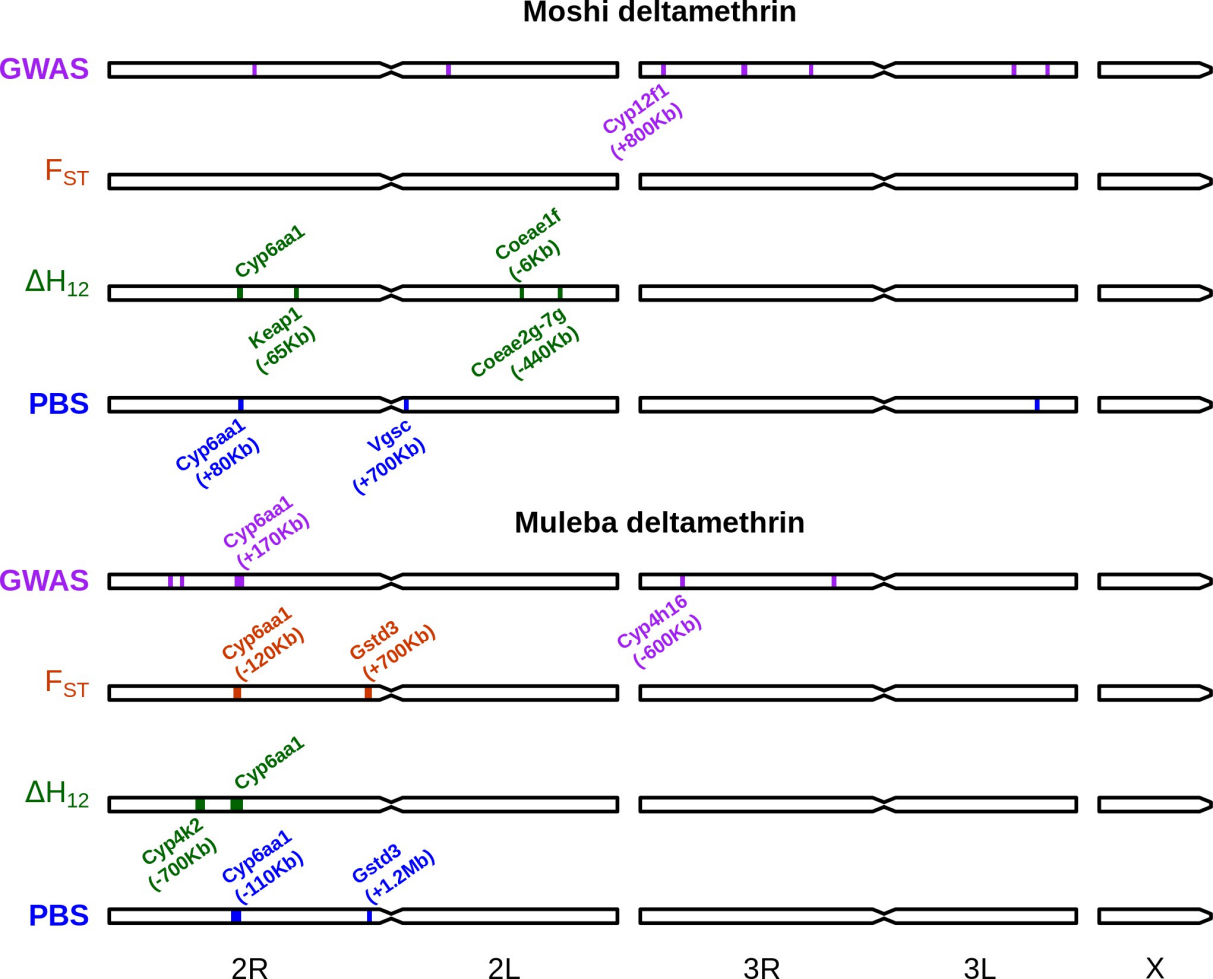
Genomic regions implicated in deltamethrin resistance according to our 4 approaches (windowed GWAS, F_ST_, ΔH_12_, PBS). Regions are annotated with genes discussed in the manuscript as possibly causing the signal. Genomic distances in brackets indicate the distance of the peak either to the left (-) or right (+) of the gene in question. The scripts and data for this figure can be found in the GiuHub repository https://github.com/vigg-lstm/GAARD_east (https://zenodo.org/records/13898157). This figure was produced using the script manuscript_figures/Figure2.r. The underlying data can be found at misc_scripts/GAARD_SNP/summary_figures/classical_analysis_snp_clump_regions_tanzania.csv, haplotypes/haplotype_significance_tests_tanzania.csv, randomisations/H12/h12_filtered_windows_tanzania.RDS and randomications/PBS/pbs_filtered_windows_tanzania.RDS. GWAS, genome-wide association study.

We performed agnostic genomic scans of phenotype association as described for a previous analysis [49]. This uses F_ST_, PBS, and ΔH_12_ (difference in H_12_ signal between resistant and susceptible subsets) calculated in 1,000 SNP windows, as well as identifying 100,000 bp windows with a high frequency of low *P*-value SNPs identified with an SNP-wise GWAS (Figs 2 and I in S1 Text and S3–S6 Data). F_ST_ is a standard measure of genetic differentiation to identify any type of genetic differences between groups greater than expected by chance. PBS [51] is based on F_ST_, but is directional and uses an outgroup to add an estimate of positive selection, thus identifying windows in which resistant samples show signs of faster genetic evolution than susceptible samples. ΔH_12_ [49] identifies windows where haplotype diversity is lower in one group compared to the other, an indication of stronger selection in that group. GWAS calculates the statistical association with resistance at each SNP across the genome. Using 4 measures of differentiation provides a rough indication of confidence in the phenotypic association in a given region, based on how consistently the region is identified across the different methods.

For deltamethrin (Fig 2), the region around *Cyp6aa1* ( *Cyp6aa/Cyp6p* region) was consistently associated with resistance in both Moshi and Muleba, and across most methods, although the signal was not always centred directly on this gene cluster, sometimes being as far as 170 Kbp away. Outside of this gene region, 2 signals of association were near other clusters of cytochrome P450s ( *Cyp12f1-4* in Moshi, *Cyp4h16-18* in Muleba) and in Moshi the PBS analysis suggested a region 700 Kbp away from the target site *Vgsc* was associated with resistance. There were ΔH_12_ signals of association in Moshi near *Keap1* (AGAP003645, 65Kbp away) and with both *Coeae1f-2f* (6 Kbp away) and *Coeae2g-7g* (440 Kbp away). Given that carboxylesterases are not typically associated with pyrethroid resistance, we performed molecular docking analysis against deltamethrin and found that neither resistance through degradation nor sequestration of the insecticide could be ruled out (S7 Data). In Muleba, both PBS and F_ST_ detected regions of association with resistance near the end of chromosome 2R, which were respectively 1.2 Mbp and 700 Kbp away from the *Gstd* cluster of glutathione esterase genes. Previous work had demonstrated the presence of CNVs in *Gstd3* in *An*. *arabiensis* [52] and *Cyp12f1* in *An*. *gambiae* [53]. We therefore investigated whether copy number in these 2 genes was associated with resistance. We found elevated copy number of *Gstd3* in 5% and 3% of samples from Muleba and Moshi, respectively, and copy number was nearly significantly associated with resistance to deltamethrin in Muleba (*P* = 0.052) but not Moshi (*P* = 0.39). When combining both locations together and including location as a random effect, copy number in *Gstd3* reached marginal significance (*P* = 0.046) when it was the only fixed effect term in the model, but this significance disappeared when copy number of *Cyp6aa1* (*P* = 0.0016) was also included, leaving the association of *Gstd3* uncertain. We found no CNVs in *Cyp12f1* in *An*. *arabiensis*. Revisiting our data from West Africa as above, we did find CNVs in *Cyp12f1* in all populations, but these were not associated with resistance to either deltamethrin or PM.

For PM, there were few windows associated with resistance, and they were not close to any gene families typically associated with resistance. Interestingly, we found a window with a high frequency of low *P*-value SNPs in the region around the *Ace1* gene (340 Kbp away), despite the lack of known resistance SNPs or CNVs in *Ace1* in this population.

## 3. Discussion

We have identified a new cluster of carboxylesterase genes associated with resistance to PM, and possibly deltamethrin, in wild-caught *Anopheles* mosquitoes. A CNV encompassing the genes *Coeae2g* - *Coeae6g* was found at much higher prevalence in Moshi, where PM resistance was prevalent, compared to Muleba, where resistance was absent. Furthermore, a larger CNV in the same gene cluster in *An*. *coluzzii* from Ghana was significantly associated with survival to PM exposure. Carboxylesterases are a classic example of insecticide resistance driven by CNVs, with the genes *Est2* and *Est3* in *Culex* mosquitoes [23], E4 and FE4 in *Myzus persicae* aphids [32], and *CCEae3a* and *CCEae6a* in *Aedes* mosquitoes [30,31], showing highly elevated copy number associated with resistance to organophosphates. We similarly found very high copy number of *Coeaexg*_Dup1 in Tanzania, with as many as 26 extra copies in a single individual, yet curiously there was no significant association of copy number with PM resistance in these samples. One possibility is that the very high frequency of the CNV (being found in 93% of samples in Moshi), led to low statistical power, but we note that copy number was highly variable, ranging from 1 extra copy to 26, and we would therefore expect that this variability in copy number would be associated with resistance and provide sufficient power.

While there was no statistical association of *Coeae2g-7g* or *Coeae1f-2f* copy number with resistance to PM in either of our Tanzanian sites, our agnostic genome-wide scans found evidence of association with deltamethrin resistance near both these gene clusters in Muleba. This is unexpected given the previous lack of convincing evidence for a role of esterases in pyrethroid resistance in mosquitoes. Furthermore, in our CNV analysis, copy number of neither gene group was associated with resistance to deltamethrin. Given that the association signals were 6 Kbp away from *Coeae1f-2f*, and 440 Kbp away from *Coeae2g-7g*, it may be that these results are false positives. However, we consider that the presence of 2 independent signals in related carboxylesterases makes the possibility of false positives unlikely, and our molecular docking analysis could not rule out the possibility of deltamethrin detoxification by these proteins. Furthermore, in other insect taxa such as the cotton bollworm and green lacewing, esterases have been implicated in pyrethroid resistance [24,54,55]. These gene clusters are therefore of concern as potential causes of cross-resistance.

As has been found in *An*. *gambiae* and *An*. *coluzzii* [28,29,49], resistance to deltamethrin in *An*. *arabiensis* seems to be primarily driven by the *Cyp6aa/Cyp6p* cluster, with this being a consistent conclusion throughout our analysis, from selection scans, GWAS and CNV association studies. In *An*. *gambiae* and *An*. *coluzzii*, this metabolic resistance occurs in a context in which target site resistance is largely fixed. In contrast, in *An*. *arabiensis*, it seems to be the dominant form of resistance, a situation similar to that found in *An*. *funestus*, where P450-based resistance is widespread, with very rare target site mechanisms [15,56–59]. The CNV alleles found in *An*. *arabiensis* are distinct from those in *An*. *gambiae* and *An*. *coluzzii*, but similarly provide resistance to deltamethrin. The emergent picture from the *An*. *gambiae* species complex is thus that metabolic resistance to deltamethrin is consistently driven by mutations in the *Cyp6aa*/*Cyp6p* cluster [49,58,60], and that these mutations are very often CNVs in *Cyp6aa1*. These CNVs are however frequently accompanied by other mutations. For example, in our study, both of the CNVs that we found appear on the background of a haplotype undergoing a hard selective sweep, yet only the CNVs, not the haplotype, were significantly associated with deltamethrin resistance, suggesting that the CNVs provide a substantial boost to resistance. In *An*. *gambiae* from Uganda, Kenya, Tanzania, and the Democratic Republic of Congo, a CNV covering only *Cyp6aa1* (*Cyp6aa*_Dup1), again associated with deltamethrin resistance, has spread to near fixation over the course of around 10 years [28]. In a striking parallel with our study, this CNV occurs on the background of a swept haplotype, although the non-CNV version of this haplotype is now so rare that phenotypic analysis of the CNV in isolation from other mutations on the haplotype cannot be performed.

This repeated combination of CNV and non-CNV resistance mutations across *Anopheles* species provides a striking parallel to what is seen in *Drosophila melanogaster*, where DDT resistance is provided by repeated mutations in another *Cyp6* gene, *Cyp6g1*, in which CNVs and transposable element insertions combine to produce highly resistant haplotypes [61]. The orthology of individual *Cyp6* genes between *Anopheles* and *Drosophila* is unclear, but our evidence suggests a consistent pattern of evolutionary genetic processes governing metabolic resistance to insecticides, with multiple mutation types accumulating to produce increasing levels of expression. We should therefore expect to see this pattern repeated in other species and other loci. As well as being an interesting illustration of the genomic mechanisms of rapid contemporary adaptation, this also creates a challenge for future genetic monitoring. When a first adaptive mutation appears on a wild-type background, the ensuing spread of the mutant creates a selective sweep where linked neutral mutations also increase in frequency, leaving a detectable drop in haplotype diversity and many mutations that can be used as correlated resistance markers. When subsequent mutations appear on an existing swept haplotype, there may be no other mutations around them to form new sweeps, making them more difficult to detect through the windowed analyses employed here. These additional mutations may therefore go unnoticed without continued targeted study of these regions.

In Ag1000g, a total of 38 CNVs have now been described at the *Cyp6aa/Cyp6p* locus, although many are rare or have not yet been tested for resistance association [62]. Over and above this huge diversity of CNVs, other non-CNV mutations are either confirmed or suspected to bring about resistance. In Ghana, a swept haplotype in the *Cyp6aa*/*Cyp6p* cluster was associated with resistance to deltamethrin [49]. While a CNV was found in the cluster, it did not include *Cyp6aa1* and was not associated with resistance to deltamethrin. In Cameroon, a non-CNV haplotype has been shown to be associated with pyrethroid resistance [63], while 2 large signals of selection are found around the same gene cluster [47], in the absence of any CNVs. While these haplotypes have not yet been phenotypically tested, we believe it very likely that they are associated with deltamethrin resistance, given the consistent results coming out of our study and the wider literature.

Resistance mutations in the deltamethrin target site, *Vgsc*, were very rare in our data, with only 2 samples in Moshi carrying the *Vgsc*-995F mutation. Strikingly, these 2 *Vgsc*-995F haplotypes were of different evolutionary origins and have not introgressed into *An*. *arabiensis* from *An*. *gambiae*. *Vgsc*-995F has been consistently present but rare in Moshi over the 10 years preceding our collections [41,64]. Our results suggest that the mutation has independently originated twice in *An*. *arabiensis* and been under sufficient selective pressure to persist in the population, but not to reach high frequency, despite pyrethroid-driven evolution evidenced by the presence of P450-based metabolic resistance. Mutations in this codon as a pyrethroid resistance mechanism are taxonomically very widespread, from mosquitos and flies to aphids and cockroaches [9–13], and thus the scarcity of these mutations in resistant populations such as ours, or as in *An*. *funestus* [59] is puzzling. One possibility is that the benefits of target site resistance to pyrethroids are lower in *An*. *arabiensis* than in *An*. *gambiae s*.*s*. and *An*. *coluzzii*, or that the physiological costs of such resistance are higher. However, the high frequency of *Vgsc*-995 mutations in *An*. *arabiensis* from West Africa suggests that target site resistance can be maintained in this species. The explanation for these differences may lie in the evolutionary history of these populations, their past exposure to DDT (which has the same target site, but different metabolic resistance pathways) and the order in which target site and metabolic resistance first appeared in the population.

Our agnostic genome-wide scans also revealed an association with deltamethrin resistance around 3 other detoxification loci: 2 cytochrome P450 clusters (*Cyp4h16-Cyp4h18* and *Cyp12f1*-*Cyp12f4*) and a glutathione-S-transferase cluster (*Gstd*). *Cyp4h17*, a member of the first cytochrome P450 cluster, was highlighted as one of the most strongly up-regulated genes in a genome-wide meta-analysis of resistant *Anopheles* expression data [65], suggesting a role in resistance, which our data indicate could be against deltamethrin. In the *Cyp12f* cluster, *Cyp12f2* and *Cyp12f3* showed allelic imbalance in gene expression in F1 crosses between resistant and susceptible colonies of *An*. *gambiae*, suggesting differential *cis* regulation of expression linked to resistance [66]. Furthermore, the presence of *Cyp12f1* CNVs in both *An*. *gambiae* and *An*. *coluzzii* also hints at a possible role of this gene in resistance. When we originally described CNVs genome-wide in these 2 species [53], we listed all cytochrome P450s, glutathione-S-transferases and carboxylesterases in which a CNV had been found. All of the genes in this list are in gene clusters that had previously, or have since, been shown to play a role in insecticide resistance in *Anopheles*, with the largest exception being *Cyp12f1*, which was only known for the possible association of *Cyp12f* genes with bendiocarb resistance in *An*. *gambiae* from Cameroon [67]. It appears that all the metabolic genes in which we had identified CNVs have now accumulated evidence of association with resistance, suggesting that the presence of CNVs in such genes should in and of itself be considered as likely predictive evidence of a role in insecticide resistance.

As with *Cyp4h17*, *Gstd3* was also highlighted as consistently differentially expressed in resistant field populations compared to laboratory colonies in a transcriptomic meta-analysis [65], and we further showed equivocal evidence of an association of copy number of this gene with resistance to deltamethrin in our data. Further evidence is needed to determine the importance of this gene in resistance and the insecticides to which resistance is most strongly conferred.

A conspicuous absence of signal in our data was in the region of *Cyp9k1* on chromosome X, which in *An*. *gambiae* and *An*. *coluzzii* showed evidence of association to both deltamethrin and PM [68–70].

Our results also provide evidence of resistance to PM in *An*. *arabiensis* from Tanzania. We found resistance in only one site, Moshi, while in Muleba there was full susceptibility, despite PM-based IRS having been applied there 3 years before our sampling [3]. Given the predominance of farming in Moshi, exposure to agricultural pesticides may be the cause of the elevated resistance to PM in this region. This exposure may have been more pervasive, over a longer period of time, and may therefore have been more effective at driving the evolution of resistance than IRS in outdoor-biting species such as *An*. *arabiensis*.

Further work based on these results should follow 2 primary lines of enquiry. First, functional genetic work is needed to quantify the resistance effects of the genes and mutations which we have identified. This includes overexpression of the cytochrome P450s, esterases and glutathione S transferases, as well as knock-ins of the CNVs. We suggest a particular focus on the *Coeae1f-2f* and *Coeae2g-7g* esterase clusters because of the current scarcity of known mutations for metabolic resistance to PM, because of the puzzling contrast in phenotypic effect between *Anopheles* species, and because of the possibility of a cross-resistance effect to pyrethroids. The great diversity of *Cyp6aa*/*Cyp6p* CNVs also warrants functional investigation to better understand the variation in effect and the determinants of their importance. Second, the putative markers of resistance that we have identified should be incorporated into molecular surveillance platforms to monitor their evolution and spread. For metabolic resistance, this is challenging because the causative mutations are either CNVs (which are difficult to genotype using high-throughput genotyping platforms as they primarily target small sequence variation) or are yet to be identified. For the purposes of monitoring, SNP markers that tag the resistance-associated haplotypes (but are not necessarily directly causative) could be used as a proxy. We have previously suggested that, given the challenge of genetically monitoring such a diverse landscape of resistance mutations in gene clusters such as *Cyp6aa*, more general methods, such as measuring gene expression directly, should be researched and developed to complement genetic screening panels [49]. We add to this the suggestion that including a measure of copy number for key gene clusters (e.g., *Cyp6aa*, *Coeae1g-7g*, *Coeae1f-2f*) in resistance monitoring activities would present an alternative solution which, while encompassing fewer resistance mutations, would perhaps come with fewer challenges. This could be achieved through digital PCR (accurate but high cost and requiring more specialised equipment) or qPCR (less accurate but lower cost and more widespread).

## 4. Methods

### 4.1. Sample collection and resistance characterisation

Mosquito larvae were collected from June to August 2018 from 2 locations in Tanzania, Moshi [−3.384, 37.349] and Muleba [−2.092, 31.574] (S1 Data). Moshi is an irrigated agricultural area south of Mount Kilimanjaro where *An*. *arabiensis* has historically been the primary vector of malaria [41]. Bednet distribution campaigns may have created selective pressure, although resistance levels to pyrethroids are generally moderate [41]. Muleba is on the border of Lake Victoria and has been the site of IRS campaigns since 2007, and of vector control trials involving bednets and IRS. The village in which we collected our samples (Kyamyorwa) was targeted with lambda-cyhalothrin IRS from 2007 to 2011, bendiocarb IRS in 2011 to 2012 [71] and PM IRS from 2014 to 2017 [72]. Historically, *An*. *gambiae s*.*s*. has been the dominant malaria vector in the region, but the intervention trials have resulted in a large reduction in numbers for that species, and the preponderance of *An*. *arabiensis* [72].

**Fig 3:**
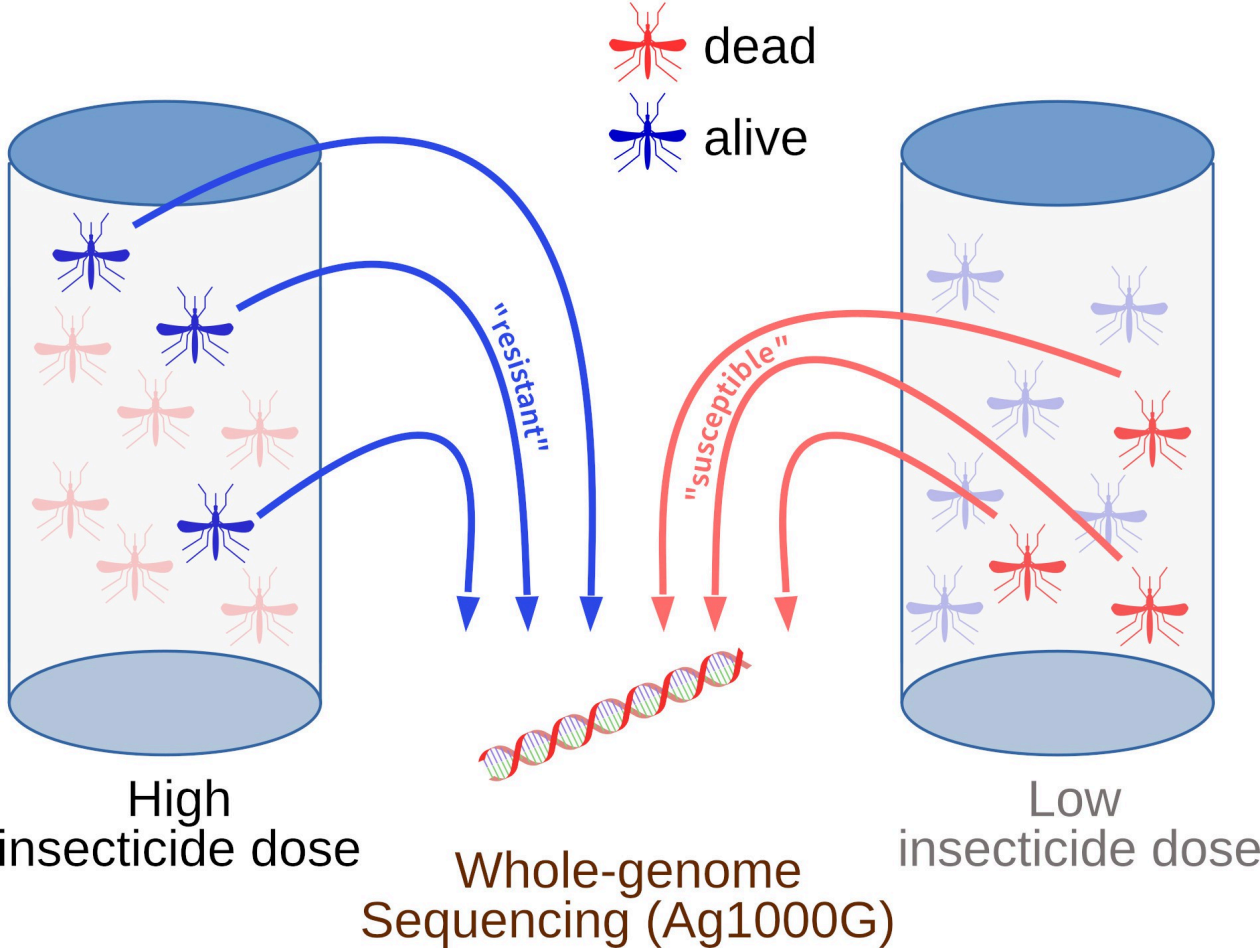
Summary of phenotyping protocol to obtain good separation of resistant and susceptible phenotypic groups for whole genome sequencing.

Mosquito larvae collected from Moshi and Muleba were respectively transported for rearing to the insectary at the National Institute for Medical Research (NIMR), Amani Centre and the NIMR Mwanza insectary, and 3- to 5-day-old females were characterised for resistance to insecticides (deltamethrin or PM) using our previously described method [49]. Briefly, we first performed a dose-response experiment to establish lethal doses to each insecticide in each location, and then identified susceptible mosquitoes as ones that were killed by a relatively low dose of insecticide, and resistant mosquitoes as ones surviving a relatively high dose (Fig 3). This created greater phenotypic separation between susceptible and resistant samples, and thus greater power to detect significant associations. The results of the dose-response experiment, the doses used for each insecticide/location, sampling locations and dates, list of specimens and molecular species identification [73,74] are available in S1 Data.

### 4.2. Whole genome sequencing and bioinformatic analysis

Overall, 489 samples were sequenced by the Ag1000G (full details of the pipeline: https://malariagen.github.io/vector-data/ag3/methods.html). Sample QC removed 2 samples for cross-contamination (*alpha* > 4.5%), 15 samples for low coverage (coverage <10× or less than 50% of genome with coverage >1×), 4 samples as apparent technical replicates, and 1 sample for unclear sex calling. A total of 467 samples passed QC filtering. SNP, CNV, and phased haplotype data were released as part of Ag1000G release 3.7. All analyses using SNPs were performed using only loci that passed Ag1000G site quality filters.

We used 2 aspects of the Ag1000G CNV calls [53,75], https://malariagen.github.io/vector-data/ag3/methods.html#cnv-calling: gene copy number based on sequencing coverage (the amount of sequencing data from a given genomic region is in proportion to the number of copies of that region in the sample) and detection of known CNV alleles using discordant reads (the point where one copy of the repeated region ends and the next begins, known as the CNV breakpoint, produces discordant sequencing reads unique to each CNV allele). Ag1000G calculates sequencing coverage state in 300 bp windows across the genome and applies a hidden Markov model (HMM) to these data to estimate the most likely copy number state in each window. Copy number of a given gene is then calculated as the modal copy number state across all the windows in the gene. Individual CNV alleles are associated with discordant reads (read pairs mapping facing away from each other or in the same direction and soft-clipped reads) consistently found at the start and end points of each CNV, allowing alleles to be identified and matched between samples (https://www.malariagen.net/data, [33]). We investigated CNVs in 6 regions with previously known association with insecticide resistance (*Ace1*, *Cyp6aa/Cyp6p*, *Cyp6m/Cyp6z*, *Gste*, *Coeae1f/Coeae2f*). In the *Cyp6aa/Cyp6p*, *Coeae1f-2f*, and *Coeae2g-7g* regions, the HMM indicated the presence of CNVs in samples without known CNV alleles. We described these CNVs by manually identifying discordant reads consistently found in the samples in which these alleles were present. These diagnostic reads will allow detection of these alleles in other whole genome sequencing data sets. The new CNVs have now been integrated into the Ag1000G CNV screening pipeline, and details of their start and end points can be found in S2 Data.

Copy number of individual genes was calculated as the mode of the HMM state within each gene. In the case of *Coeaexg*_Dup1, copy number often far exceeded the maximum copy number state allowed in the HMM (10 extra copies). To obtain a more accurate value of copy number for this CNV, we instead took the median raw normalised coverage for all windows found within the CNV region (positions 37282000 to 37295000) and subtracted 2 (the normal diploid copy number). Thus, for this CNV, copy number was calculated across *Coeae2g - Coeae6g*, rather than for each gene independently.

The KING statistic of kinship [46] was calculated using NGSRelate [76] using genome-wide SNPs, excluding regions of genomic inversions (2L,13-38Mb and 2R,19-33Mb). We used a threshold KING value of 0.185 (S1 Methods) to classify full sibs. From each sib group, we randomly chose a single individual to retain for all analyses described below, discarding the others. The exception to this was the calculation of F_ST_, where it was computationally feasible to permute which sibs were removed (see below).

Selection scans were performed using H_12_ [77] and H_1X_ [48]. H_1X_ is a measure of haplotype sharing between 2 populations, calculated as $H_{1X}=\sum_{i=1}^{n}\;(H_{ia}.H_{ib})$, where *n* is the number of haplotypes found in either population, and *H*_*ia*_ and *H*_*ib*_ are the frequencies of haplotype *i* in populations *a* and *b*, respectively. High values of H_1X_ indicate that high frequency (i.e., swept) haplotypes are shared between the 2 populations.

### 4.3. Identification of swept haplotypes and association with CNVs

Clusters of highly similar haplotypes, indicative of a selective sweep, were determined by hierarchical clustering on pairwise genetic distance (Dxy) between haplotypes, producing a tree as in Fig 1D, where the tips of the tree (at Dxy = 0) represent haplotypes, and the genetic distance between any 2 haplotypes is approximated by the height on the y axis reached by their connecting path. Long horizontal lines (as seen on the right of Fig 1D) thus indicate a group of identical haplotypes as found in a selective sweep.

CNV calls are not phased and therefore only available at the level of the individual mosquito, not at the level of each of the 2 haplotypes found in each mosquito. Therefore, CNV status cannot be directly ascribed to each haplotype node in the clustering tree. We instead identified SNPs that were highly correlated with CNV allele calls at the mosquito level (correlation coefficients for *Coeaexg*_Dup1, *Coeaexf*_Dup2, and *Cyp6aap*_Dup33 were equal to 1, 0.87, and 0.92, respectively) and used these SNPs as a proxy for CNV presence at the haplotype level, allowing CNV status of each node to be indicated (purple bars in Fig 1D). In the case of *Cyp6aap*_Dup31 (Fig F in S1 Text), no suitable proxy SNP could be found, and we instead indicate the number of copies of the CNV found in the individual from which each haplotype was derived. Full workings to reproduce this analysis can be found at https://github.com/vigg-lstm/GAARD_east/blob/main/CNV_analysis/sweeps.

### 4.4. Phenotypic association of CNVs and known resistance SNPs

We investigated association between resistance phenotype and individual genetic markers (CNVs or SNPs) using generalised linear models (glm) implemented in R v4 [78], with binomial error and a logit link function, with phenotype as the dependent variable and genotypes as independent variables. SNP genotypes were coded numerically as the number of mutant alleles (possible values of 0, 1, and 2), CNV alleles were coded as presence/absence, and gene copy number was coded as the number of extra copies. Starting from the null model, we proceeded by stepwise model building, adding the most highly significant marker at each step until no remaining markers provided a significant improvement.

To calculate the statistical power of finding an effect of *Coeae1f* on resistance to PM, we took the data in which a significant association had previously been found [33] and calculated that mortality had been 44.2% in wild-type individuals and 16.7% in individuals carrying a CNV. Mortality in wild-type individuals was almost the same in Moshi (44.4%). We therefore ran 1,000 Monte Carlo simulations using our sample size and CNV frequency from Moshi, with the mortalities observed in Ghana. For each randomisation, we ran the same glm as on the real data and calculated the proportion of simulations in which we observed *P* < 0.05.

### 4.5. Agnostic genome-wide analysis of resistance association

We performed genome-wide scans for association with resistance using 4 measures of genetic differentiation between resistant and susceptible samples (F_ST_, PBS, ΔH_12_, GWAS).

We calculated F_ST_ using the *moving_patterson_fst* function in *scikit-allel* [79] in a moving window of 1,000 SNPs, after filtering SNPs for missing data and removing singletons (SNPs present only once in the data set). In order to take advantage of the full sample set despite nonindependence of siblings, we performed permutations in which one randomly chosen individual per sib group was used in the calculation of F_ST_. In Muleba, we performed 100 such permutations and calculated the mean F_ST_ of all permutations. In Moshi, the PM sample set contained no sibs, while the deltamethrin sample set contained only 1 pair of sibs and thus needed only averaging the 2 calculations of F_ST_ (removing each sib in turn).

Provisional windows of interest (“peaks”) were identified as ones with positive F_ST_ values 3 times further from the mode than the smallest negative value [49]. We then removed peaks that might be the result of the presence of a selective sweep, as opposed to true association of that sweep with resistance [49], using Monte Carlo simulations creating 500 permutations of the phenotype labels and recalculating F_ST_ for each permutation. We retained peaks whose observed F_ST_ was greater than 99% of the simulations.

F_ST_ indicates any genetic differences between resistant and susceptible samples, but we expect that differences associated with resistance would be associated with the presence of swept haplotypes at higher frequency in the resistant compared to susceptible samples. To further filter the F_ST_ peaks, we therefore explored the presence of swept haplotypes within each peak. Haplotype clusters were determined by hierarchical clustering (section 4.3), and cutting the tree at a height of 0.001. Clusters comprised of at least 20 haplotypes were tested for association with phenotype using a generalised linear model with binomial error and logit link function, with phenotype as the response and sample genotype (number of copies of the haplotype) as a numerical independent variable. Peaks were discarded if they did not contain a haplotype positively associated with resistance.

We calculated H_12_ using the *garuds_h* function in *scikit-allel* in a moving window of 1,000 SNPs, using phased biallelic SNPs. The ΔH_12_ metric was obtained by subtracting H_12_ in the susceptible samples from H_12_ in the resistant samples, with a positive value thus indicating a higher frequency of swept haplotypes in resistant samples. PBS between susceptible and resistant samples was calculated using segregating SNPs in 1,000 SNP windows using the *pbs* function in *scikit-allel*, with *An*. *arabiensis* samples from Malawi, collected in 2015, as the outgroup (Ag1000G phase 3.0 data release). As with ΔH_12_, positive signals of PBS indicate stronger positive selection in the resistant samples. We identified provisional peaks in PBS by taking windows with a PBS value higher than 3 times the 95th centile of the PBS distribution. Using this threshold for H_12_ resulted in a very large number of provisional peaks across the entire genome, and we thus used 3 times the 98th centile as a threshold instead for H_12_. For both H_12_ and PBS, 500 Monte Carlo permutations of phenotype were performed as above to remove false positive peaks caused by the presence of swept haplotypes.

We performed SNP-wise GWAS using SNPs with no missing data and a minor allele count of at least 5. In a previous study, we found that contamination of our samples by *Asaia* bacteria caused artefacts in our association analysis [49]. We therefore used Bracken [80] to estimate the amount of *Asaia* contamination in each sample and excluded SNP loci where genotype was correlated with *Asaia* levels (*P* < 0.05).

For each SNP, we used a GLM with binomial error and logit link function to obtain a *P*-value of association for phenotype against genotype (coded as the number of non-reference alleles). We used *fdrtool* [81] to perform false-discovery rate correction, with *Q* value threshold of 1%. We also used these data to perform a windowed analysis, identifying the 1,000 most significant SNPs in each sample set and looking for 100,000 bp windows that contained at least 10 SNPs among the top 1,000.

## Supporting information

S1 Data Bioassay results, sample metadata and sample CNV calls. (XLSX)

S2 Data Start and end points of CNV allele ranges. (XLSX)

S3 Data Breakdown of genome-wide F_ST_ analysis results. (HTML)

S4 Data Breakdown of genome-wide ΔH12 analysis results. (HTML)

S5 Data Breakdown of genome-wide PBS analysis results. (HTML)

S6 Data Breakdown of genome-wide association study results. (HTML)

S7 Data Methods and results of molecular docking analysis. (PDF)

S1 Text Supplementary figures and tables. (PDF)

S1 Methods Supplementary methods. (PDF)

## Abbreviations

CNV: copy number variant
GAARD: Genomics for African Anopheles Resistance Diagnostics
GWAS: genome-wide association study
HMM: hidden Markov model
IRS: indoor residual spraying
ITN: insecticide-treated net
NIMR: National Institute for Medical Research
PCA: principal component analysis
PM: pirimiphos-methyl
SNP: single-nucleotide polymorphism
