# Supplementary material for: Copy number variants underlie major selective sweeps in insecticide resistance genes in Anopheles arabiensis
Source: PLoS Biol. 2024 Dec 5;22(12):e3002898. doi: 10.1371/journal.pbio.3002898 (PMC11620391; doi:10.1371/journal.pbio.3002898)
Supplement: S7 Data — (PDF) [file pbio.3002898.s007.pdf]

Molecular docking of deltamethrin with carboxylesterases

To assess the potential metabolism of deltamethrin by the implicated carboxylesterases, we performed a series of molecular docking experiments. We first downloaded predicted protein models from the AlphaFold database, and predicted their interactions with ligand models of the deltamethrin. For molecular docking we used software GNINA (McNutt *et al.*, 2021), a method which applies autodock vina for docking followed by pose scoring with a convolutional neural network (CNN). GNINA outputs the predicted binding affinities between the receptor and ligand, as well as a CNN score - a value between 0 and 1 which can be seen as a probability of a pose likely to be observed in nature.

We first ran molecular docking with a ligand acting as a positive control, 4-nitrophenyl butyrate, a commonly used model substrate for esterase enzymes (Levisson, van der Oost and Kengen, 2009). After docking with GNINA and assessment of the top 10 predicted binding poses (Table A below), all esterases had multiple predicted poses with CNN scores of greater than 0.7, in conjunction with multiple poses with low inter-molecular distances (< 4Å) between the nucleophilic serine and carbonyl of the ester bond. This indicated that, as expected, these carboxylesterases are likely to bind and metabolise 4-nitrophenyl butyrate.

We then examined the top 10 predicted binding poses of deltamethrin with each carboxylesterase putatively implicated in resistance. *Coeae1f* was predicted to interact with deltamethrin with moderate binding affinities (-6.6 to -7.9 kcal/mol, Table A, Fig. A & B). Importantly, however, these were all in pose conformations in which the distance from the nucleophilic Serine's hydroxyl group to the carbonyl group of the deltamethrin ester bond was greater than 6Å. This distance is extremely unlikely to lead to ester cleavage and metabolism (Torrance *et al.*, 2007). These pose predictions were accompanied by low values in the CNN score (0.27-0.49) reported by GNINA, implying that they are unlikely to be productive poses in which metabolism may occur. In contrast, *Coeae2f* showed stronger binding affinities with deltamethrin (-7.4 to -9.0 kcal/mol) and three conformations in the top 10 poses led to serine-ester bond distances of around 4Å, including the highest-ranked pose which had a distance of 3.9Å and a CNN score of 0.83. For this pose, BINANA (Durrant and McCammon, 2011) predicts a hydrogen bond from the nitrile group of deltamethrin to the active-site serine of *Coeae2f* and π–π stacking between the benzyl ring and a tryptophan residue, which may contribute to metabolism by helping to position the ligand in a productive conformation. Overall, these results suggest that *Coeae2f* may bind to deltamethrin in a conformation conducive to metabolism.

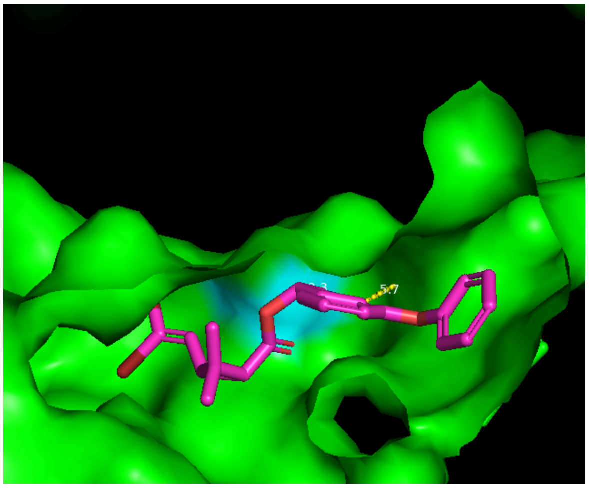

Figure A. The substrate binding pocket of AGAP0006228 (*Coeae2f*) docked with deltamethrin in a productive pose. The nucleophilic Serine is highlighted in turquoise.

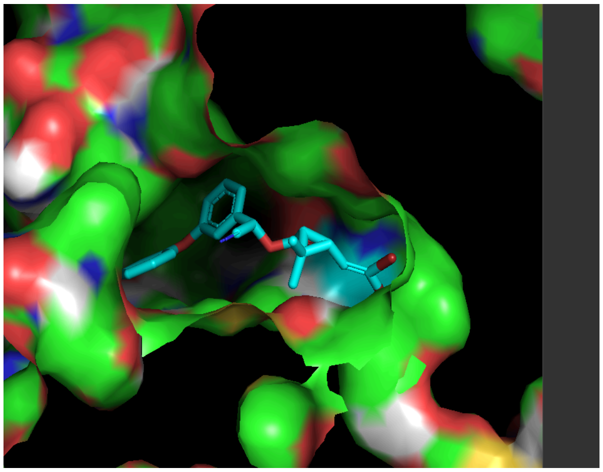

Figure B. The substrate binding pocket of AGAP0006724 (*Coeae3g)* docked with deltamethrin in a productive pose. The nucleophilic Serine is highlighted in turquoise.

The *Coeae2g-7g* cluster genes show varied docking profiles against deltamethrin. The second-ranked pose of deltamethrin predicted interaction with *Coeae2g* (AGAP006723) had a strong binding affinity of -8.00 Kcal/Mol, as well as a low distance of 3.15Å between the Serine hydroxyl group and deltamethrin ester bond, a pose which could potentially lead to cleavage of the ester bond. In contrast, *Coeae3g* (AGAP006724) displays strong binding affinities for deltamethrin (-7.79 to -8.92 Kcal/Mol) and high CNN scores but mostly large distances between the serine and deltamethrin ester bond (4.76 to 8.25Å), which could suggest that sequestration may be a more likely mechanism of action than direct metabolism for this enzyme. Predicted poses for *Coeae3h* (AGAP006725), *Coeae5g* (AGAP006726), and *Coeae6g* (AGAP006727) all included some with strong binding affinities and low distances between the serine and deltamethrin ester bond, again suggesting that metabolism by these enzymes could be possible.

Methods

We downloaded predicted protein models (.pdb format) from the Alphafold database (Jumper *et al.*, 2021; Varadi *et al.*, 2024). Insecticide ligands were downloaded from Pubchem in the 3D conformation (.sdf format). SDF ligands were converted to .pdbqt and protonated with OpenBabel (O'Boyle *et al.*, 2011). We first detected the active site of each carboxylesterase by searching for the esterase motif '[LIV].G.S.G' within the amino acid coding sequence, and

locating the hydroxyl group of the nucleophilic serine of the esterase catalytic triad. The coordinates of the hydroxyl group are input to the molecular docking software GNINA (McNutt *et al*., 2021), with which we centre a grid search box of 20 x 20 x 20 Ångströms. GNINA is a fork of autodock vina (Trott and Olson, 2010), and uses a convolutional neural network to score predicted ligand poses. The top 10 predicted ligand poses were then evaluated by calculating the distance from the nucleophilic hydroxyl group to the ester bonds of deltamethrin, followed by visual assessment of the ligand in the substrate binding pocket. Finally, we explored receptor-ligand binding with the tool BINANA (Durrant and McCammon, 2011). Code used to run molecular docking experiments can be found at https://github.com/vigg-lstm/GAARD_east/blob/main/carboxylesterase_docking.

Table A. Results of molecular docking of carboxylesterase genes with deltamethrin and 4-nitrophenyl-butyrate-ester (positive control)

Binding pose rank	Affinity (kcal / mol)	Intramol (kcal / mol)	CNN_score	CNN_affinity (kcal / mol)	Distance (Å)	ligand	gene
1	-6.21	-0.14	0.7606	4.702	2.93	4-nitrophenyl-butyrate-ester	AGAP006227
2	-5.21	-0.3	0.7059	4.537	3.88	4-nitrophenyl-butyrate-ester	AGAP006227
3	-4.61	-0.07	0.7028	4.424	10.45	4-nitrophenyl-butyrate-ester	AGAP006227
4	-5.63	-0.28	0.5338	4.75	3.27	4-nitrophenyl-butyrate-ester	AGAP006227
5	-4.91	-0.11	0.4855	4.237	4.56	4-nitrophenyl-butyrate-ester	AGAP006227
6	-4.96	-0.25	0.4306	4.285	10.11	4-nitrophenyl-butyrate-ester	AGAP006227
7	-5.9	-0.04	0.4038	4.737	6.86	4-nitrophenyl-butyrate-ester	AGAP006227
8	-5.33	0.02	0.4012	4.434	8.92	4-nitrophenyl-butyrate-ester	AGAP006227
9	-4.99	1.04	0.3962	4.153	6.53	4-nitrophenyl-butyrate-ester	AGAP006227
1	-6.07	-0.25	0.8772	4.971	3.61	4-nitrophenyl-butyrate-ester	AGAP006228
2	-6.21	-0.34	0.8742	5.094	9.01	4-nitrophenyl-butyrate-ester	AGAP006228
3	-6.24	-0.15	0.87	4.957	3.13	4-nitrophenyl-butyrate-ester	AGAP006228
4	-5.57	-0.28	0.7061	4.523	6.73	4-nitrophenyl-butyrate-ester	AGAP006228
5	-5.76	-0.17	0.6828	4.591	5.45	4-nitrophenyl-butyrate-ester	AGAP006228
6	-4.98	-0.48	0.679	4.943	9.55	4-nitrophenyl-butyrate-ester	AGAP006228
7	-6.16	-0.21	0.65	4.636	3.79	4-nitrophenyl-butyrate-ester	AGAP006228
8	-5.29	-0.09	0.6261	4.345	3.32	4-nitrophenyl-butyrate-ester	AGAP006228
9	-5.44	-0.26	0.6233	4.434	4.64	4-nitrophenyl-butyrate-ester	AGAP006228
1	-7.09	0.06	0.7873	5.3	7.23	4-nitrophenyl-butyrate-ester	AGAP006723
2	-5.54	-0.37	0.7682	5.171	4.27	4-nitrophenyl-butyrate-ester	AGAP006723
3	-6.18	-0.29	0.7154	5.058	6.24	4-nitrophenyl-butyrate-ester	AGAP006723
4	-7	0.14	0.7017	5.038	7.24	4-nitrophenyl-butyrate-ester	AGAP006723
5	-6.99	0.15	0.6892	5.026	7.29	4-nitrophenyl-butyrate-ester	AGAP006723
6	-5.55	-0.15	0.6527	4.968	3.15	4-nitrophenyl-butyrate-ester	AGAP006723
7	-4.53	-0.21	0.6413	4.985	8.39	4-nitrophenyl-butyrate-ester	AGAP006723

8	-5.23	-0.13	0.5955	4.965	3.48	4-nitrophenyl-butyrate-ester	AGAP006723
9	-6.68	-0.19	0.585	4.873	3.77	4-nitrophenyl-butyrate-ester	AGAP006723
1	-6.34	0.18	0.9395	5	6.92	4-nitrophenyl-butyrate-ester	AGAP006724
2	-6.21	-0.4	0.8507	4.897	7.12	4-nitrophenyl-butyrate-ester	AGAP006724
3	-6.16	-0.41	0.8439	4.884	7.18	4-nitrophenyl-butyrate-ester	AGAP006724
4	-5.5	-0.31	0.8412	4.881	4.42	4-nitrophenyl-butyrate-ester	AGAP006724
5	-6.1	-0.37	0.8047	4.85	6.36	4-nitrophenyl-butyrate-ester	AGAP006724
6	-6.29	0.2	0.7916	4.838	6.52	4-nitrophenyl-butyrate-ester	AGAP006724
7	-5.31	-0.25	0.7824	4.825	3.04	4-nitrophenyl-butyrate-ester	AGAP006724
8	-5.48	-0.24	0.7789	4.615	3.09	4-nitrophenyl-butyrate-ester	AGAP006724
9	-5.22	-0.12	0.7453	4.708	5.90	4-nitrophenyl-butyrate-ester	AGAP006724
1	-6.41	0.06	0.8703	5.168	4.30	4-nitrophenyl-butyrate-ester	AGAP006725
2	-6.27	0.08	0.8007	5.255	3.82	4-nitrophenyl-butyrate-ester	AGAP006725
3	-6.59	-0.28	0.7819	5.181	8.39	4-nitrophenyl-butyrate-ester	AGAP006725
4	-0.74	-0.19	0.7215	4.893	2.94	4-nitrophenyl-butyrate-ester	AGAP006725
5	-6.29	-0.04	0.7162	5.006	4.21	4-nitrophenyl-butyrate-ester	AGAP006725
6	-6.3	0.29	0.6485	4.634	5.56	4-nitrophenyl-butyrate-ester	AGAP006725
7	-4.59	-0.25	0.6125	4.876	4.14	4-nitrophenyl-butyrate-ester	AGAP006725
8	-5.66	-0.35	0.6068	4.327	8.17	4-nitrophenyl-butyrate-ester	AGAP006725
9	-6.38	0.91	0.5951	4.859	5.57	4-nitrophenyl-butyrate-ester	AGAP006725
1	-6.42	-0.36	0.8455	5.066	3.04	4-nitrophenyl-butyrate-ester	AGAP006726
2	-6.39	-0.28	0.8199	4.981	3.09	4-nitrophenyl-butyrate-ester	AGAP006726
3	-6.3	-0.28	0.7939	5.078	3.57	4-nitrophenyl-butyrate-ester	AGAP006726
4	-4.98	-0.45	0.71	4.726	4.02	4-nitrophenyl-butyrate-ester	AGAP006726
5	-4.59	0.11	0.6574	4.564	3.31	4-nitrophenyl-butyrate-ester	AGAP006726
6	-5.3	-0.16	0.6568	4.62	6.63	4-nitrophenyl-butyrate-ester	AGAP006726
7	-6.32	-0.39	0.6543	4.806	4.93	4-nitrophenyl-butyrate-ester	AGAP006726
8	-4.96	0.36	0.6482	4.836	3.87	4-nitrophenyl-butyrate-ester	AGAP006726
9	-5.71	-0.26	0.5338	4.683	4.11	4-nitrophenyl-butyrate-ester	AGAP006726
1	-5.27	-0.21	0.858	5.137	6.39	4-nitrophenyl-butyrate-ester	AGAP006727
2	-5.64	-0.3	0.8499	4.997	3.20	4-nitrophenyl-butyrate-ester	AGAP006727
3	-5.59	-0.11	0.8328	5.143	3.12	4-nitrophenyl-butyrate-ester	AGAP006727
4	-6.12	0.39	0.8322	5.109	6.91	4-nitrophenyl-butyrate-ester	AGAP006727
5	-5.64	-0.28	0.8262	5.089	3.20	4-nitrophenyl-butyrate-ester	AGAP006727
6	-4.79	-0.32	0.7964	4.997	3.89	4-nitrophenyl-butyrate-ester	AGAP006727
7	14.22	-0.05	0.7821	4.883	2.76	4-nitrophenyl-butyrate-ester	AGAP006727
8	-5.08	-0.43	0.7086	4.98	7.35	4-nitrophenyl-butyrate-ester	AGAP006727
9	-5.12	-0.24	0.707	4.978	3.71	4-nitrophenyl-butyrate-ester	AGAP006727
1	-7.89	0.01	0.4886	6.362	9.62	deltamethrin	AGAP006227
2	-6.76	-0.1	0.4825	6.618	7.96	deltamethrin	AGAP006227
3	-6.87	0.67	0.4758	6.65	7.42	deltamethrin	AGAP006227
4	-7.69	-0.26	0.4312	6.512	7.69	deltamethrin	AGAP006227

5	-6.47	-0.36	0.4025	6.705	6.56	deltamethrin	AGAP006227
6	-6.59	-0.06	0.3991	6.48	7.49	deltamethrin	AGAP006227
7	-7.16	0.2	0.3049	6.246	6.81	deltamethrin	AGAP006227
8	-7.36	0.04	0.2979	5.801	9.77	deltamethrin	AGAP006227
9	-7.15	0.25	0.2777	6.184	6.64	deltamethrin	AGAP006227
1	-8.29	2.76	0.8305	7.242	3.91	deltamethrin	AGAP006228
2	-7.92	1.46	0.7498	7.177	4.12	deltamethrin	AGAP006228
3	-8.46	0.85	0.7312	7.362	3.45	deltamethrin	AGAP006228
4	-8.76	-0.78	0.7245	6.989	6.01	deltamethrin	AGAP006228
5	-9	-0.9	0.6862	6.961	6.66	deltamethrin	AGAP006228
6	-8.12	0.48	0.6803	7.104	5.93	deltamethrin	AGAP006228
7	-8.71	-0.02	0.6278	6.918	5.71	deltamethrin	AGAP006228
8	-7.49	0.51	0.5961	6.87	5.91	deltamethrin	AGAP006228
9	-7.36	-0.37	0.5763	6.927	4.79	deltamethrin	AGAP006228
1	-7.35	0.91	0.4667	7.006	4.32	deltamethrin	AGAP006723
2	-8	-0.05	0.4645	6.988	3.16	deltamethrin	AGAP006723
3	-6.92	0.32	0.4345	6.132	7.17	deltamethrin	AGAP006723
4	-5.56	1.82	0.4258	6.749	5.11	deltamethrin	AGAP006723
5	-6	1.22	0.4217	6.771	4.89	deltamethrin	AGAP006723
6	-5.16	2.01	0.409	6.803	5.12	deltamethrin	AGAP006723
7	-8.18	-0.1	0.4086	6.78	4.11	deltamethrin	AGAP006723
8	-7.75	0.69	0.408	6.952	3.10	deltamethrin	AGAP006723
9	-7.41	0.03	0.4057	6.79	4.80	deltamethrin	AGAP006723
1	-8.92	-0.39	0.8698	6.95	7.30	deltamethrin	AGAP006724
2	-8.61	0.27	0.7838	7.005	4.76	deltamethrin	AGAP006724
3	-8.89	0.83	0.7768	6.831	7.08	deltamethrin	AGAP006724
4	-8.02	0.3	0.7548	6.948	8.26	deltamethrin	AGAP006724
5	-7.95	1.9	0.7505	7	6.52	deltamethrin	AGAP006724
6	-8.75	0.12	0.746	6.855	7.59	deltamethrin	AGAP006724
7	-8.55	0.56	0.7152	6.968	6.70	deltamethrin	AGAP006724
8	-7.79	1.24	0.7094	6.855	6.31	deltamethrin	AGAP006724
9	-8.87	0.15	0.7047	6.823	6.60	deltamethrin	AGAP006724
1	-7.06	0.3	0.7621	7.57	3.80	deltamethrin	AGAP006725
2	-3.33	0.85	0.6768	7.547	4.85	deltamethrin	AGAP006725
3	-5.24	1.84	0.672	7.415	2.99	deltamethrin	AGAP006725
4	-7.21	0.62	0.6695	7.134	11.04	deltamethrin	AGAP006725
5	-6.96	0.08	0.6661	7.445	3.73	deltamethrin	AGAP006725
6	-6.96	0.19	0.6196	7.526	3.70	deltamethrin	AGAP006725
7	-7.27	-0.01	0.5992	6.97	10.20	deltamethrin	AGAP006725
8	-4.37	1.59	0.5961	7.356	2.59	deltamethrin	AGAP006725
9	-2.18	-0.19	0.5915	7.09	5.70	deltamethrin	AGAP006725
1	-7.32	7.37	0.6699	7.119	6.13	deltamethrin	AGAP006726

2	-8	-0.04	0.6675	7.243	3.41	deltamethrin	AGAP006726
3	-8.16	0.24	0.6454	7.338	3.34	deltamethrin	AGAP006726
4	-7.46	-0.31	0.6372	6.969	6.49	deltamethrin	AGAP006726
5	-6.67	0.17	0.6331	7.013	6.16	deltamethrin	AGAP006726
6	-7.64	-0.22	0.6277	6.833	3.53	deltamethrin	AGAP006726
7	-6.67	5.1	0.6176	7.015	8.39	deltamethrin	AGAP006726
8	-7.79	-0.08	0.6151	6.933	5.41	deltamethrin	AGAP006726
9	-8.21	0.25	0.6095	6.968	6.45	deltamethrin	AGAP006726
1	-7.29	3.43	0.6373	7.299	4.13	deltamethrin	AGAP006727
2	-6.8	-0.39	0.6191	7.161	6.02	deltamethrin	AGAP006727
3	-6.87	0.01	0.6042	7.289	4.91	deltamethrin	AGAP006727
4	-7.57	0.23	0.6019	7.134	5.63	deltamethrin	AGAP006727
5	-5.65	1.71	0.5618	7.092	5.16	deltamethrin	AGAP006727
6	-5.1	0.57	0.5356	7.01	3.96	deltamethrin	AGAP006727
7	12.38	0.93	0.51	6.387	4.60	deltamethrin	AGAP006727
8	-5.17	3.27	0.5072	6.924	4.46	deltamethrin	AGAP006727
9	-3.03	1.3	0.5051	7.016	6.63	deltamethrin	AGAP006727

References:

Durrant, J.D. and McCammon, J.A. (2011) ‘BINANA: a novel algorithm for ligand-binding characterization’, *Journal of molecular graphics & modelling*, 29(6), pp. 888–893.

Jumper, J. *et al.* (2021) ‘Highly accurate protein structure prediction with AlphaFold’, *Nature*, 596(7873), pp. 583–589.

Levisson, M., van der Oost, J. and Kengen, S.W.M. (2009) ‘Carboxylic ester hydrolases from hyperthermophiles’, *Extremophiles: life under extreme conditions*, 13(4), pp. 567–581.

McNutt, A.T. *et al.* (2021) ‘GNINA 1.0: molecular docking with deep learning’, *Journal of cheminformatics*, 13(1), p. 43.

O’Boyle, N.M. *et al.* (2011) ‘Open Babel: An open chemical toolbox’, *Journal of cheminformatics*, 3(1), p. 33.

Torrance, J.W. *et al.* (2007) ‘The geometry of interactions between catalytic residues and their substrates’, *Journal of molecular biology*, 369(4), pp. 1140–1152.

Trott, O. and Olson, A.J. (2010) ‘AutoDock Vina: improving the speed and accuracy of docking with a new scoring function, efficient optimization, and multithreading’, *Journal of computational chemistry*, 31(2), pp. 455–461.

Varadi, M. *et al.* (2024) ‘AlphaFold Protein Structure Database in 2024: providing structure

coverage for over 214 million protein sequences’, *Nucleic acids research*, 52(D1), pp. D368–D375.
