# Supplementary material for: Copy number variants underlie major selective sweeps in insecticide resistance genes in Anopheles arabiensis
Source: PLoS Biol. 2024 Dec 5;22(12):e3002898. doi: 10.1371/journal.pbio.3002898 (PMC11620391; doi:10.1371/journal.pbio.3002898)
Supplement: S1 Methods — (PDF) [file pbio.3002898.s009.pdf]

Copy number variants underlie major selective sweeps in insecticide resistance genes in *Anopheles arabiensis*.

Electronic Supplementary Material

Supplementary methods

Choice of kinship treshold

We calculated pairwise kinship between all samples using the KING statistic (Manichaikul et al. 2010) implemented in NGSRelate (Korneliussen and Moltke 2015) using SNP data across the whole genome after masking inversions. Results indicated a positive bias in kinship, with the mode of the distribution above 0 (Fig. **M1**). Because of this positive bias in kinship values, we sought to empirically establish the most parsimonious threshold to identify full siblings in our data, instead of the threshold of 0.177 suggested in the manual (`https://www.kingrelatedness.com/manual.shtml`). For all possible threshhold between 0.15 and 0.35, in increments of 0.005, we identified all full siblings and counted the proportion of full sib groups that contained inconsistencies (where siblings of siblings were not themselves classed as siblings). We chose the threshold 0.185 as the most stringent threshold that did not result in incomplete sib groups (where two individuals are both sibs of a third individual, but not sibs of each-other).

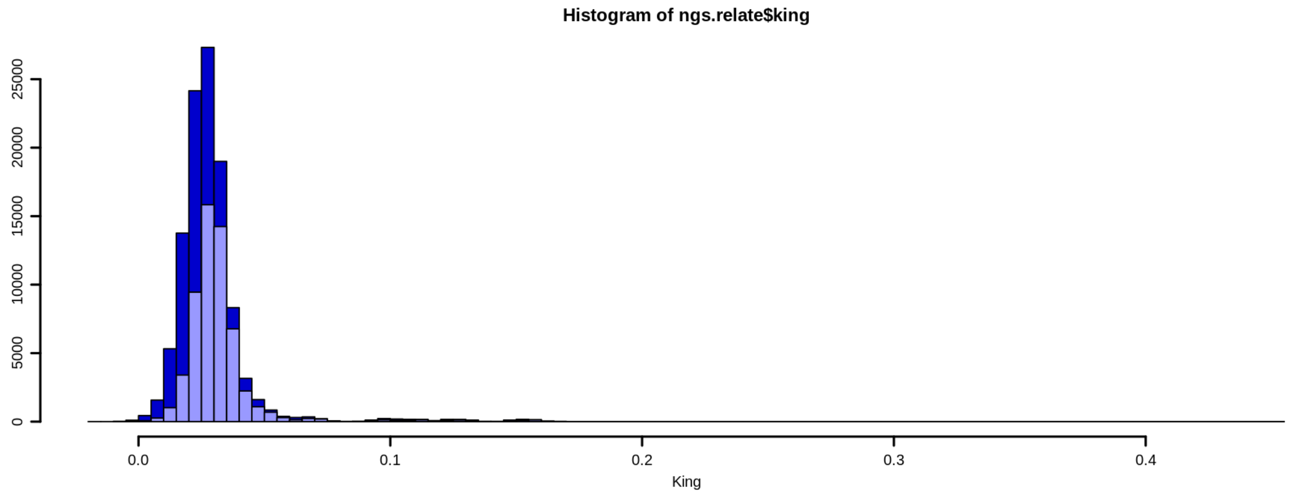

Fig. M1: Histograms of kinship values (KING scores, expected score between full sibs = 0.25, expected score between unrelated individuals = 0) across all sample pairs.

References

Korneliussen, T. S. and I. Moltke (2015). NgsRelate: a software tool for estimating pairwise relatedness from next-generation sequencing data. *Bioinformatics* 31(24):4009–4011.

Manichaikul, A. et al. (2010). Robust relationship inference in genome-wide association studies. *Bioinformatics* 26(22):2867–2873.
